# Supplementary material for: The Norwegian Microbiota Study in Anorexia Nervosa (NORMA): Integrating a clinical trial with preclinical experiments–A study protocol
Source: PLoS One. 2026 Mar 11;21(3):e0342275. doi: 10.1371/journal.pone.0342275 (PMC12978472; doi:10.1371/journal.pone.0342275)
Supplement: S2 File — (PDF) [file pone.0342275.s002.pdf]

Norwegian University of Life Sciences

§ 8. Research protocol NORMA study - Gut flora and anorexia nervosa

a) **Project Manager:**

Professor Siv Kjølsrud Bøhn, Norwegian University of Life Sciences (NMBU).

Participating researchers:

Siv Kjølsrud Bøhn, NMBU - Norwegian University of Life Sciences
Øyvind Rø, Oslo University Hospital HF Regional Division of Eating Disorders RASP
Harald Carlsen, NMBU - Norwegian University of Life Sciences
Bjørge Westereng, NMBU - Norwegian University of Life Sciences
Knut Rudi, NMBU - Norwegian University of Life Sciences
Cynthia Bulik Karolinska Institutet, Stockholm, Sweden

Collaborators:

Oslo University Hospital HF Regional Division of Eating Disorders RASP by Øyvind Rø
Karolinska Institutet in Stockholm by Cynthia Bulik
Modum Bad ved Johan Dahl
Nord-Trøndelag Regional Health Authority by Siri Weider
Helse Bergen HF Haukeland University Hospital at Ute Kessler
Nordland Hospital Trust, represented by Gro Anita Ytterstad
Innlandet Hospital by Geir Rune Nyhus
The Eating Disorder Association at Rooy Rodríguez Ramírez
Counselling on eating disorders by Line Orvedal

b) a scientifically designed project plan stating the project's purpose, rationale, material, methods, substantiation that the chosen study design can provide an answer to the research question and estimated time frames for the project

Purpose of the project:

The overall goal of the NORMA (**Norwegian Microbiota A**norexia Nervosa) project is to increase our understanding of the role that the gut flora plays in the physical and mental symptoms of anorexia. The specific objectives of the project are to find out what distinguishes the gut flora in anorexia patients from normal-weight controls and whether the gut flora is normalized in anorexia patients when they undergo standard treatment at an eating disorder clinic. In the project, we will also investigate how the interaction between diet and gut flora affects appetite, intestinal problems and other anorexia-associated problems during treatment. Furthermore, preclinical laboratory experiments will be conducted with intestinal flora from anorexia patients to identify new types of prebiotics (carbohydrate types) that can potentially be used to normalize the intestinal flora in the patients.

The project consists of 3 work packages (WP-1-3). In WP I, we will conduct a clinical trial to a) compare the gut flora between healthy controls and patients with severe anorexia b) investigate the interaction between diet and gut microbiota and find out if symptoms such as low appetite, gastrointestinal problems and other anorexia-associated problems can be explained by the composition of the gut flora c) Investigate whether standard treatment normalizes the gut flora in patients with severe anorexia d) Investigate the degree of recurrence of eating disorders and mental illness at long-term follow-up over 10 years.

In WP2 and WP 3, preclinical studies of the gut flora from patients with anorexia will be performed.

In WP2, we will perform a laboratory experiment where we will test the effect of different types of prebiotics on the gut flora extracted from the faeces of anorexia patients. The goal is to identify prebiotics that can normalize the gut flora in anorexia patients.

In WP3, an animal fecal transplant experiment will be carried out in which faeces from anorexia patients are transferred to mice. The aim is a) to establish an anorexia-mouse model in which the anorexia symptom picture with lower weight gain and a higher degree of anxiety and obsessive-compulsive disorder and then b) test whether the symptom picture can be reversed by supplementation of the prebiotic types identified in WP 2.

Reason:

Anorexia nervosa is a serious mental disorder that particularly affects young women. The disorder has major consequences for those affected, where quality of life, participation in working life and fertility are affected. All these consequences contribute to major personal, family and societal costs. Treatment of anorexia often involves a combination of nutritional treatment and different types of psychotherapeutic approaches. But the treatment is complicated and only 50 percent of patients get satisfactory results [1]. In addition, medical complications are common due to underweight, and mortality is high [2]. Therefore, there is a great need for new strategies for anorexia treatment. In addition, there is no information basis for evaluating the long-term effects of the gut flora on the course of anorexia. We therefore want to retain the opportunity to collect information on medication use and readmission for eating disorders and/or mental illness and to send requests for new stool samples for gut flora analyses after 5 and 10 years.

Methods:

Design: The study is a multicenter cross-sectional study, with a longitudinal design where a patient group is followed over time. A control group will be measured at one point in time and used as a reference.

Trial population: Patients who will be admitted to a ward for the treatment of severe with anorexia will be asked to participate in the study. Patients who follow standard treatment at the clinic they are admitted to will be followed from admission to discharge, a period lasting about 12 weeks. The control group will be recruited among healthy normal weight to mildly obese volunteers and data/biomaterial will be collected at one point in time.

Recruitment: The collaborating eating disorder clinics will contribute to the recruitment of the patients to the study. We will follow a three-point strategy for recruitment. 1) Advance information, 2) Reflection time, 3) Re-contact and request for participation.

1) Advance information: In advance, upon invitation to the prevention meeting, the patients have received an email with a link to the study's website where they can find information, both information videos and written information. The study is presented to the patient for the first time at the end of the first preventive care meeting. The guardianship meeting can be either physical or digital. 2) Reflection time: Patients will then be asked to think about whether they want to participate. They will receive (or be sent if the meeting is digital) written information about the study and sampling equipment for possible collection and sending of stool samples. They will also be sent links to video information about the study by email. 3) Re-contact and request for participation: At the second meeting, which can be either physical or digital, patients are asked if they would like to participate and if they have any questions. If they agree to participate, they must submit informed consent. This is done digitally via a link that is sent to the patient by SMS, or email. The participant will receive guidance along the way. After recruitment and signing of consent, the patient receives specific information about what to do.

The controls are primarily recruited from the Eastern Norway area via intranet websites at NMBU/OUS. We expect that even if the controls have a place of residence and study/place of work in Eastern Norway, they will still be representative of upbringing in different geographical areas in Norway.

Stool samples: Stool samples (1-3 tubes) must be collected 3 times during the study: 1) In the week prior to admission (by the patient himself in a home situation). 2) After 6 weeks of hospitalization, 3) On discharge (about 12 weeks after admission). The first sample should be taken by the patient himself at home. Instructions are available both in writing, on video and by phone follow-up. Tests 2 and 3 are taken after admission. All stool samples to be used for microbiota sequencing are taken using a method (Roche Diagnostics Stool Transport and Recovery (STAR) buffer (Fisher Scientific, Hampton, NH, USA) which means that the sample can be administered by the patient either at home (Trial 1) or during admission (Trials 2 and 3). This collection method ensures that the DNA in the samples is stable for many days at room temperature and can thus withstand being sent by mail to NMBU in envelopes for biological material. The disadvantage, however, is that the STAR-buffer method cannot be used for transplantation into mice (WP3) or for the ex vivo experiment (WP2). For such use, the stool sample must be treated within a short time with a protocol adapted for faecal transplantation before freezing. This will require trained laboratory personnel. Samples for WP2 and 3 will therefore only be collected from a sample of the patients from RASP and a selection of the controls administered by NMBU. For individuals who contribute samples to all WPs, we will therefore be retrieved in two different ways, i.e. 1) with the STAR buffer method and 2) without the addition of STAR buffer, prepared for fecal transplantation.

Blood samples: In addition to the blood samples taken as part of hospitalization, only one extra serum tube will be taken for the NORMA study at three time points (baseline, 6 weeks and about 12 weeks) for biobanking at NMBU. The patients will then follow the standard follow-up at the individual clinic. The

procedures at the various clinics are somewhat different (see table). For example, standard follow-up is shown for the RASP clinic. The results from blood test results taken as part of admission and treatment at the clinics will be extracted from patient records and used as part of the NORMA study. Typical clinical biomarkers that are measured are: Metabolic biomarkers (serum), liver biomarkers (AST/ALT) (serum), hemoglobin (EDTA), cell diff (EDTA), iron status (serum), electrolytes (serum), creatinine (serum), CRP (serum). The healthy controls will either take blood samples at the Fürst laboratory or by a research employee at NMBU who is authorized to take blood samples. The blood samples will be analyzed for the same clinical biomarkers as the patients. In addition, serum from 2 tubes will be biobanked in a research biobank for the NORMA study.

Urine samples: No additional urine samples will be collected for the NORMA study, but the results of clinical analysis of urine samples requested as part of standard of care will be made available to the study.

Data collection via form.

Cost registrations. Prior to admission, patients must make a 3-day dietary registration via a web-based solution (see assessment under research ethics challenges) that will reflect the participants' baseline diet before admission. Participants will receive follow-up over phone/video call to complete this. If, for various reasons, the participant is unable to do so prior to admission, both the stool sample and the dietary registration will be carried out during the first week as an alternative plan. The diet is recorded using a diet diary in the same week as blood samples and stool samples are taken. In order not to burden the participants more than necessary, we will use household measurements rather than weighted cost registration. Uploading food photos will only be an option for the control population and for the patients who register their diet prior to admission, as photography/use of mobile phones is not allowed during the meal situation at the clinics. Macro- and micronutrient intake will be calculated using a cost calculation program (KBS) developed at the University of Oslo.

Physical activity Registered with a few simple questions in connection with the cost registrations. We plan to include physical activity questions from NORDIET-FFQ [3] that have been validated.

Forms for registration of **relevant background** (AN history, antibiotic use, use of supplements and medications, demographics, height and weight) will be completed only at baseline.

Questionnaires for **anorexia and extent** (EDEQ), **anxiety** (GAD-7), **depression** (PHQ9), **obsessive-compulsive disorder** (OCI-R), **quality of life** (SF36) and **GI complaints** (ROMA) at baseline will also take place during the first few days after admission.

Biomarker analyses. Stool samples will be analyzed for inflammation biomarkers, biomarkers for intestinal integrity, metabolism and biomarkers relevant to gut-brain connection.

Substantiation that the chosen study design can provide answers to the research question and estimated time frames for the project:

The sample size was based on the objective of WP 1, which aims to investigate differences between the gut flora of anorexia patients compared with healthy people. The sample size was estimated by using a web page (fedematt.shinyapps.io/shinyMB)[4] for simulation-based strength calculations. The method uses

a sially Dirichlet-Multinomial model to describe and generate the presence/abundance of bacteria. We estimated that the sample size required to detect a significant difference between AN patients and HD would be 79 in each group. With an estimated non-response rate of ~10 %, we plan to include n = 90 in each group. The assumptions used for the calculation were that the default setting for the website was used, except that #OTUs were set to 100. The significance level (α) was set to 0.05 and the abundance curves were created based on the 5 most abundant OTUs increasing by 60% and the 5 second most abundant OTUs increasing by 50%.

In conversation with the collaborating clinics, we have estimated that, based on current figures (patient throughput), we will reach the number of 90 patients within about 1.5 years to 2 years from the start of the study.

c) how health information is to be processed, including from which sources health information is to be collected and whether such information is to be disclosed to others or transferred to countries outside the EEA

Collection of health information and processing

A digital battery will be used to collect self-reported data on the basis (history of anorexia disease, use of antibiotics, use of supplements and medications, demographics, height and weight), on anorexia disease (ED100K/**EDE-Q** and Clinical impairment questionnaire (**CIA 3.0),** GI symptoms (GSRS-IBSe.l), 4-day dietary registration, depression (PHQ-9 or similar), anxiety (GAD-7), obsessive-compulsive disorder (OCI-R), and quality of life (SF36). All data will be collected using an integrated sensitive data collection (Nettskjema.no) solution established and operated by TSD (https://www.uio.no/english/services/it/research/sensitive-data/). In addition, a web form will be established for registration of relevant clinical information from medical records that will be used by the project staff member in the study. Each participant is given an anonymous ID number when recruiting for the study. The code key is stored in a separate access-regulated folder on TSD. Only the project manager (Siv Kjølsrud Bøhn, NMBU) and the medical manager (Øyvind Rø) have access to the code key in TSD. A different ID number (Id2) will be used for partners named under point a) for the duration of the study. This number will be used when labeling the biomaterial collected in the study. Data generated from analysis of biomaterial at NMBU will be stored on secure servers at NMBU and uploaded to TSD for linking to id1 and other data collected. Pseudo-identified data from TSD will be downloaded to secure servers at NMBU for statistical handling. A data management plan has been drawn up for the project and submitted to the Research Council as part of the funded project (Appendix, Data management plan version 1.0). The plan is intended to be a dynamic document that will be updated to new versions as the project is implemented.

In addition to data collected as part of the study and patient records, we want access to data from the following registers:

Requested information	Register	0-1 years	5 years	10 years
Patient background	NORSPIS **(including SCL-90-R, EDE-Q 6.0, CIA 3.0)**	x		

Drug treatment of<br>• Mental disorders<br>• Sleep<br>• Antibiotic use<br>• Hormonal treatment	**The Norwegian Medicines Registry** is a health registry with information about prescription drugs dispensed in pharmacies.	**x**	**x**	**x**
Admission for anorexia or other eating disorder and/or mental illness.	**Norwegian Patient Registry NPR**	**x**	**x**	**x**
Admission for anorexia or other eating disorder and/or mental illness.	**KUHR (Control and Payment of Health Reimbursements)** is a system that handles reimbursement claims from treatment providers and health institutions to the state (HELFO).	**x**	**x**	**x**

Sharing of health information

Involved partners must sign a valid agreement on data and material transfer before any data and/or material is transferred. The purpose of such an agreement is to regulate rights and obligations in accordance with the Personal Data Act and the General Data Protection Regulation (the Data Protection Authority).

Sharing health information with other countries

Health information must not be sent out of the country.

d) the sources from which human biological material is to be extracted and whether such material is to be disclosed to others or transferred abroad;

In addition to sample collection that is included in the standard assessment upon admission and treatment in the clinic, an additional serum tube will be collected for biobanking and later analysis of other biomarkers. The sample will be divided into 4 alliquots, of which 2 will be stored in the Research Biobank for the NORMA study. From the controls, two tubes will be taken for direct analysis of clinical biomarkers at Fürst as well as two serum tubes that will be stored in the research biobank for the NORMA study (see section on extended consent). The serum samples are planned to be analyzed at laboratories in Norway, preferably at Oslo University Hospital and NMBU.

The stool samples will be stored in the research biobank for the NORMA study (see section on extended consent). The samples will be used for biomarker analyses and for mapping of gut flora based on isolated microbial DNA and subsequent sequencing. In addition, a fecal graft is prepared for WP2 and 3 from a selection of the patient samples and controls. Primarily, we plan that laboratory preparation of material for sequencing and sequencing will take place at NMBU, but if it is economically expedient to purchase this service from external suppliers in Norway or abroad, such as Oslo Sequencing Center, DNAsense (Denmark), Eurofins (Germany), Ziel (Germany) or Novogene (UK), we want to have the opportunity to do this. In the event of a transfer to a foreign laboratory, the samples will only be marked with anonymous ID numbers, and REC will be informed if we enter into such an agreement with a detailed description of the procedure for material and data transfer and agreements related to this.

e) assessment of research ethics challenges associated with the project, in particular the benefit-risk aspect for research participants

Apart from stool collection, a couple of extra blood tests and the dietary examination/completion of the form, the patient participants will not be exposed to any significantly greater burden than they are exposed to during follow-up/treatment at the ward for eating disorders. Overall, we believe that the benefits of the project are proportionate to the risk/disadvantage to which the patients are exposed. For the controls, the conclusion is the same. The check-ups will gain deeper insight into their own diet and good advice on how they can eat healthier. Feedback on the diagnostic blood values will also be a useful health check.

There are several ethical issues associated with intervention trials with food in anorexia populations since interference with standard treatment can have unexpected and undesirable effects. The use of the preclinical methods in the current project therefore takes these issues into account and makes it possible to study the gut flora in an ex vivo setting where it can be experimentally manipulated.

The two associations for eating disorders, SPISFO and ROS, which are partners in the study, have participated in several planning meetings for the study and have provided input to the research protocol and information letter to the participants. Thus, the user perspective has been thoroughly taken into account in the planning of the study.

f) sources of funding, interests and dependencies, including any financial circumstances of researchers and research participants related to the research project in question

The project is funded by the Research Council of Norway (Project number 336239). The controls will be informed that they will be in the draw for one (out of 5) gift card of 1000 kr. The patient participants in the study will be compensated with a gift card of NOK 250 per measurement time. Due to the modest amount of the amount and the low probability of winning, we consider that the financial appreciation will not significantly affect the decision to participate. None of the participating researchers have a financial interest in the project.

g) a plan for the publication of results and information on possible extended use, including commercial use, of research results, data or biological material.

Plan for publication of results: The results from the project will primarily be published as articles through traditional scientific channels. The first three articles will be part of a PhD degree funded by the Research Council of Norway. We will also communicate the results of the study through the Norwegian microbiota organization ReMicS (https://microbiota.no/remics/) and via websites at Oslo University Hospital and NMBU. We will create a separate website for the NORMA study operated by NMBU. The two user organisations will also contribute to popularising the results and contributing to effective communication of the results to their users. In addition, we will aim for a broad coverage of published results in social media and TV/radio. The project manager has already been interviewed by NRK.no and NRK radio (EKKO) after the allocation of research funding, had a presentation at the women's health

conference organized by the Research Council of Norway (December 2022) and has a standing invitation to be interviewed on the TV program God Morgen Norway to talk about the project.

Information about possible extended use: We want to retain the possibility of some extended use of data and material obtained in the study for the patient group and the controls during the study period. The participants in the study are therefore asked whether, in addition to participating in the NORMA study, they will give broad consent for material and data to be included in a general biobank with associated health registry (GUTBRAIN). The purpose of GUTBRAIN is that biomaterial and data from studies that investigate the gut-brain axis can be collected for comparison between different populations beyond anorexia as well as for experimental studies that can increase the understanding of the gut flora's influence on the gut-brain axis.

1 Steinhausen, H. C. The outcome of anorexia nervosa in the 20th century. *Am. J. Psychiatry* **159**, 1284-1293 (2002).

2 Murray, S. B., Quintana, D. S., Loeb, K. L., Griffiths, S. & Le Grange, D. Treatment outcomes for anorexia nervosa: a systematic review and meta-analysis of randomized controlled trials. *Psychol. Med.* **49**, 535-544, doi:10.1017/S0033291718002088 (2019).

3 Henriksen, H. B. *et al.* Validation of two short questionnaires assessing physical activity in colorectal cancer patients. *BMC sports science, medicine & rehabilitation* **10**, 8, doi:10.1186/s13102-018-0096-2 (2018).

4 La Rosa, P. S. *et al.* Hypothesis testing and power calculations for taxonomic-based human microbiome data. *PloS one* **7**, e52078, doi:10.1371/journal.pone.0052078 (2012).
