# Supplementary material for: The Norwegian Microbiota Study in Anorexia Nervosa (NORMA): Integrating a clinical trial with preclinical experiments–A study protocol
Source: PLoS One. 2026 Mar 11;21(3):e0342275. doi: 10.1371/journal.pone.0342275 (PMC12978472; doi:10.1371/journal.pone.0342275)
Supplement: S3 File — (PDF) [file pone.0342275.s003.pdf]

§ 8. Forskningsprotokoll NORMA studien -Tarmflora og anorexia nervosa

a) **Prosjektleder:**

Professor Siv Kjølsrud Bøhn, Norges miljø- og biovitenskapelige universitet (NMBU).

Deltagende forskere:

Siv Kjølsrud Bøhn, NMBU - Norges miljø- og biovitenskapelige universitet
Øyvind Rø, Oslo universitetssykehus HF Regional avd for spiseforstyrrelser RASP
Harald Carlsen, NMBU - Norges miljø- og biovitenskapelige universitet
Bjørge Westereng, NMBU - Norges miljø- og biovitenskapelige universitet
Knut Rudi, NMBU - Norges miljø- og biovitenskapelige universitet
Cynthia Bulik Karolinska Institutet, Stockholm, Sverige

Samarbeidspartnere:

Oslo universitetssykehus HF Regional avd for spiseforstyrrelser RASP ved Øyvind Rø
Karolinska Institutet i Stockholm ved Cynthia Bulik
Modum Bad ved Johan Dahl
Helse Nord-Trøndelag HF ved Siri Weider
Helse Bergen HF Haukeland universitetssjukehus ved Ute Kessler
Nordlandssykehuset HF ved Gro Anita Ytterstad
Sykehuset Innlandet ved Geir Rune Nyhus
Spiseforstyrrelsesforeningen ved Rooy Rodriguez Ramirez
Rådgivning om spiseforstyrrelser ved Line Orvedal

b) en vitenskapelig utformet prosjektplan med angivelse av prosjektets formål, begrunnelse, materiale, metoder, sannsynliggjøring av at valgt studiedesign kan gi svar på forskningsspørsmålet og anslåtte tidsrammer for prosjektet

Prosjektets formål:

Den overgripende målsetningen for NORMA (**Nor**wegian **M**icrobiota **A**norexia Nervosa) prosjektet er å øke vår forståelse for hvilken rolle tarmfloraen spiller for det fysiske og psykiske symptombildet ved anoreksi. De konkrete målsetningene ved prosjektet er å finne ut hva som skiller tarmfloraen hos anoreksipasienter fra normalvektige kontroller og om tarmfloraen normaliseres hos anoreksipasienter når de gjennomgår standard behandling ved spiseforstyrrelsesklinikk. I prosjektet skal vi også undersøke hvordan samspillet mellom kosthold og tarmfloraen påvirker appetitt, tarmproblemer og andre anoreksi-assosierte problemer under behandlingen. Videre skal det gjennomføres prekliniske laboratorieforsøk med tarmflora fra anoreksipasienter for å identifisere nye typer prebiotika (karbohydrat-typer) som potensielt kan brukes til å normalisere tarmfloraen hos pasientene.

Prosjektet består av 3 arbeidspakker (WP-1-3). I WP I skal vi gjennomføre en klinisk studie for a) Sammenlikne tarmfloraen mellom friske kontroller og pasienter med alvorlig anoreksi b) undersøke samspillet mellom kosthold og tarmmikrobiota og finne ut om symptomer som lav appetitt, gastrointestinale problemer og andre anoreksi-assosierte problemer henger kan forklares ut fra tarmfloraens sammensetning c) Undersøke om standard behandling normaliserer tarmfloraen hos pasienter med alvorlig anoreksi d) Undersøke grad av tilbakefall av spiseforstyrrelse og psykisk sykdom ved langtidsoppfølging over 10 år.

I WP2 og WP 3 skal det utføres prekliniske studier av tarmfloraen fra pasienter med anoreksi.

I WP2 skal vi utføre et laboratorieforsøk der vi skal teste effekten av ulike typer prebiotika på tarmfloraen som er hentet ut fra avføringen til anoreksipasientene. Målet er å identifisere prebiotika som kan normalisere tarmfloraen hos anoreksipasientene.

I WP3 skal det utføres et dyreforsøk med fekal transplantasjon der avføring fra anoreksi-pasienter overføres til mus. Målsetningen er a) å etablere en anoreksi-musemodell der anoreksi-symptombildet med lavere vektoppgang og høyere grad av angst og tvangslidelse for deretter å b) teste om symptombildet kan reverseres ved tilføring av prebiotikatypene identifisert i WP 2.

Begrunnelse:

Anoreksi (anoreksia nervosa) er en alvorlig psykisk lidelse som særlig rammer unge kvinner. Lidelsen har store konsekvenser for dem som rammes, der livskvalitet, deltagelse i arbeidsliv og fertilitet påvirkes. Alle disse konsekvensene bidrar til store personlige-, familiære- og samfunnsmessige kostnader. Behandling av anoreksi innebærer gjerne en kombinasjon av ernæringsbehandling og ulike typer psykoterapeutiske tilnærminger. Men behandlingen er komplisert og bare 50 prosent av pasientene får tilfredsstillende resultat [1]. I tillegg er det vanlig med medisinske komplikasjoner på grunn av undervekt, og dødeligheten er høy [2]. Derfor er det et stort behov for nye strategier for anoreksibehandling. I tillegg eksisterer det et ingen informasjonsgrunnlag for evaluering av tarmfloraens langtidseffekter på forløpet for anoreksi. Vi ønsker derfor å beholde muligheten til å innhente opplysninger om medisinbruk og reinnleggelse for

spiseforstyrrelse og/eller psykisk sykdom og for å sende forespørsel om nye avføringsprøver for tarmfloraanalyser etter 5 og 10 år.

Metoder:

Design: Studien er en multisenter-tverrsnittstudie, med longitudinelt design der en pasientgruppe følges over tid. En kontrollgruppe vil måles på ett tidspunkt og brukes som referanse.

Forsøkspopulasjon: Pasienter som skal legges inn på sengepost for behandling av alvorlig med anoreksi vil få forespørsel om å delta i studien. Pasientene som følger standard behandling ved klinikken de innlegges ved, vil følges fra innleggelse til utskrivelse, en periode som varer omkring 12 uker. Kontrollgruppen vil rekrutteres blant friske normalvektige til mildt overvektige frivillige og data/biomateriale vil samles inn ved ett tidspunkt.

Rekruttering: De samarbeidende spiseforstyrrelsesklinikkene vil bidra til rekruttering av pasientene til studien. Vi vil følge en tre-punkts strategi for rekrutteringen. 1) Forhåndsinformasjon, 2) Betenkningstid, 3) Re-kontakt og forespørsel om deltakelse.

1) Forhåndsinformasjon: På forhånd, ved invitasjon til forvernsmøte har pasientene mottatt en epost med lenke til studiens hjemmeside der de kan finne informasjon både informasjonsvideoer og skriftlig informasjon. Studien presenteres for første gang for pasienten i slutten av første forvernsmøte. Forvernsmøtet kan være enten fysisk eller digitalt. 2) Betenkningstid: Deretter vil pasientene få beskjed om å tenke på om de ønsker å delta. De får med seg (eller tilsendt hvis møtet er digitalt) skriftlig informasjon om studien og prøvetakingsutstyr for eventuell innsamling og forsendelse av avføringsprøver. De får også tilsendt lenker til videoinformasjon om studien på epost. 3) Re-kontakt og forespørsel om deltakelse: Ved andre møte, som kan være enten fysisk eller digitalt, blir pasientene spurt om de kan tenke seg å delta og om de har noen spørsmål. Dersom de sier ja til å delta må de sende inn informert samtykke. Dette skjer digitalt via en lenke som sendes til pasienten på SMS, eller epost. Deltakeren vil få veiledning underveis. Etter rekruttering og signering av samtykke får pasienten konkret informasjon om de skal gjøre.

Kontrollene rekrutteres først og fremst fra østlandsområdet via intranettsider ved NMBU/OUS. Vi forventer at selv om kontrollene har bo og studie/arbeidssted på Østlandet vil de likevel være representative for oppvekst ved ulike geografiske områder i Norge.

Avføringsprøver: Avføringsprøver (1-3 rør) skal innhentes 3 ganger ila studien: 1) Ila uken som er i forkant av innleggelse (av pasienten selv i en hjemmesituasjon). 2) Etter 6 ukers innleggelse, 3) Ved utskriving (ca 12 uker etter innleggelse). Den første prøven skal tas av pasienten selv hjemme. Instruksjoner fås både skriftlig, på video og vha telefonoppfølging. Prøve 2 og 3 tas etter innleggelse. Alle avføringsprøvene som skal benyttes for mikrobiotasekvensering tas med en metode (Roche Diagnostics Stool Transport and Recovery (STAR) buffer (Fisher Scientific, Hampton, NH, USA) som innebærer at prøven kan administreres av pasienten selv enten hjemme (prøve 1) eller under innleggelse (prøve 2 og 3). Denne innsamlingsmetoden sikrer at DNA i prøvene er stabile i mange døgn i romtemperatur og tåler dermed å sendes i posten til NMBU i konvolutter for biologisk materiale. Ulempen er imidlertid at STAR-buffer metoden ikke kan brukes til transplantasjon til mus (WP3) eller til ex vivo forsøket (WP2). For slik bruk må avføringsprøven innen kort tid behandles med en protokoll tilpasset fekaltransplantasjon før frysing. Dette vil kreve opplært laboratoriepersonell. Prøver for WP2 og 3 vil derfor kun hentes fra et utvalg av pasientene fra RASP og et utvalg av kontrollene som administreres av NMBU. For individer som bidrar

med prøver til alle WPs vil vi derfor hentes to forskjellige måter, dvs 1) med STAR buffer metoden og 2) uten tilsetning av STAR buffer, preparert for fekal transplantasjon.

Blodprøver: Utover blodprøvene som tas som del av innleggelse vil det bli kun tatt ett ekstra serumrør til NORMA-studien ved tre tidspunkt (baseline, 6 uker og ca 12 uker) for biobanking ved NMBU. Pasientene vil deretter følge standard oppfølging ved den enkelte klinikk. Prosedyrene ved de ulike klinikkene er noe avvikende (se tabell). Eksempelvis vises standard oppfølging for RASP klinikken. Resultatene fra blodprøveresultater som tas som en del av innleggelse og behandling ved klinikkene vil hentes ut fra pasientjournal og brukes som en del av NORMA-studien. Typiske kliniske biomarkører som måles er : Stoffskiftebiomarkører (serum), leverbiomarkører (ASAT/ALAT)(serum), hemoglobin (EDTA), celle diff (EDTA), jernstatus (serum), elektrolytter (serum), kreatinin (serum), CRP (serum). De friske kontrollene vil enten ta blodprøver ved Fürst laboratorium eller av forskningsmedarbeider ved NMBU som er autorisert til å ta blodprøver. Blodprøvene vil bli analysert for de samme kliniske biomarkørene som pasientene. I tillegg vil serum fra 2 rør biobankes i forskningsbiobank for NORMA studien.

Urinprøver: Det vil ikke tas ekstra urinprøver for NORMA-studien men resultatene av klinisk analyse av urinprøver som rekvireres som ledd i standard behandling vil bli gjort tilgjengelig for studien.

Datainnhenting via skjema.

Kostregistreringer. I forkant av innleggelse skal pasientene selv foreta en 3-dagers kostregistrering via en web-basert løsning (se vurdering under forskningsetiske utfordringer) som skal reflektere deltakernes baseline-kosthold før innleggelse. Deltakerne vil få oppfølging over telefon/videosamtale for å få gjennomført dette. Dersom deltakeren av ulike årsaker ikke får til dette i forkant av innleggelse vil både avføringsprøven og kostregistreringen gjennomføres den første uken som en alternativ plan. Kostholdet registreres ved bruk av kostdagbok i samme uke som blodprøver og avføringsprøver blir tatt. For å ikke belaste deltakerne mer enn nødvendig vil vi benytte husholdningsmål fremfor veid kostregistrering. Opplasting av mat-foto vil kun være en opsjon for kontrollpopulasjonen og for pasientene som registrerer kostholdet i forkant av innleggelsen da det ikke tillates fotografering/bruk av mobiltelefoner ved måltidssituasjonen ved klinikkene. Makro og mikronæringsstoff-inntak vil beregnes vha et kostberegningsprogram (KBS) utarbeidet ved Universitetet i Oslo.

Fysisk aktivitet registreres vha noen enkle spørsmål i forbindelse med kostregistreringene. Vi planlegger å inkludere spørsmål om fysisk aktivitet fra NORDIET-FFQ [3] som er validert.

Skjema for registrering av **relevant bakgrunn** (AN historikk, antibiotikabruk, bruk av supplementer og medisiner, demografi, høyde og vekt) vil fylles ut kun ved baseline.

Skjema for **anoreksi og omfang** (EDEQ), **angst** (GAD-7), **depresjon** (PHQ9), **tvangslidelse** (OCI-R), **livskvalitet** (SF36) og **GI plager** (ROMA) ved baseline vil også foregå i løpet av de første dagene etter innleggelse.

Biomarkøranalyser. Avføringsprøver vil bli analysert mhp og senere analyser av inflammasjonsbiomarkører, biomarkører for tarmintegritet, metabolisme og biomarkører relevante for gut-brain forbindelse.

Sannsynliggjøring av at valgt studiedesign kan gi svar på forskningsspørsmålet og anslåtte tidsrammer for prosjektet:

Utvalgsstørrelsen ble basert på målsetningen i WP 1 som har til hensikt å undersøke forskjeller mellom tarmfloraen hos anoreksipasienter sammenliknet med friske. Utvalgsstørrelsen ble estimert ved å benytte en web-side (fedematt.shinyapps.io/shinyMB)[4] for simuleringsbaserte styrkeberegninger. Metoden benytter en sålalt Dirichlet-Multinomial-modell for å beskrive og generere tilstedeværelse/mengde (abundance) av bakterier. Vi estimerte at utvalgsstørrelsen som kreves for å oppdage en signifikant forskjell mellom AN-pasienter og HC vil være 79 i hver gruppe. Med et estimert frafall på ~10 % planlegger vi å inkludere n = 90 i hver gruppe. Forutsetningene som ble benyttet for utregningen var at standardinnstillingen for nettsiden ble benyttet, bortsett fra at # OTUer ble satt til 100. Signifikansnivået (α) ble satt til 0,05 og abundanskurvene ble laget basert på at de 5 mest «abundant» OTUene økte med 60 % og de 5 nest mest rikelig OTUene økte med 50 %.

I samtale med de samarbeidende klinikkene har vi estimert at vi, basert på dagens tall (pasientgjennomstrømning), vil nå antallet på 90 pasienter innen ca 1.5 år til 2 år fra studiestart.

c) hvordan helseopplysninger skal behandles, herunder fra hvilke kilder helseopplysninger skal innhentes og om slike opplysninger skal utleveres til andre eller overføres til land utenfor EØS

Innhenting av helseopplysninger og behandling

Et digitalt batteri skal benyttes for innhenting av selvrapporterte data på bakgrunn (historikk om anoreksisykdom, bruk av antibiotika, bruk av supplementer og medisiner, demografi, høyde og vekt), om anoreksisykdom (ED100K/**EDE-Q** og Clinical impairment questionnaire (**CIA 3.0)** , GI symptomer (GSRS-IBSe.l), 4 dagers kostregistrering, depresjon (PHQ-9 e.l), angst (GAD-7), tvangslidelse (OCI-R), og livskvalitet (SF36). Alle data vil innhentes vha en integrert løsning for innsamling av sensitive data (Nettskjema.no) som er etablert og drives av TSD (https://www.uio.no/english/services/it/research/sensitive-data/). Det vil i tillegg etableres et nettskjema for registrering av relevant klinisk informasjon fra medisinsk journal som vil benyttes av prosjektmedarbeider i studien. Hver deltaker får et anonymt id-nummer ved rekruttering til studien. Kodenøkkelen er lagret i en egen tilgangsregulert mappe på TSD. Det er kun prosjektleder (Siv Kjølsrud Bøhn, NMBU) og medisinsk ansvarlig (Øyvind Rø) som har tilgang til kodenøkkelen i TSD. Et annet id nummer (Id2) vil benyttes for samarbeidspartnere navngitt under punkt a) så lenge gjennomføringen av studien pågår. Dette nummeret vil anvendes ved merking av biomaterialet som samles inn i studien. Data som genereres fra analyse av biomateriale på NMBU vil lagres på sikre servere ved NMBU og lastes opp i TSD for kobling mot id1 og andre data som er samlet inn. Pseudoidentifiserte data fra TSD vil lastes ned på sikre servere på NMBU for statistisk håndtering. En datahåndteringsplan er utformet for prosjektet og innsendt til Forskningsrådet som del av det finansierte prosjektet (Vedlegg, Datahåndteringsplan versjon 1.0). Planen er ment å være et dynamisk dokument som vil oppdateres til nye versjoner etter hvert som prosjektet implementeres.

I tillegg til data som innhentes som del av studien samt pasientjournal ønsker vi tilgang til data fra følgende registre:

Ønsket informasjon	Register	0-1 år	5 år	10 år
Pasientbakgrunn	NORSPIS **(inkludert SCL-90-R, EDE-Q 6.0, CIA 3.0)**	x		
Medikamentell behandling av<br>• Psykiske lidelser<br>• Søvn<br>• Antibiotikabruk<br>• Hormonell behandling	**Legemiddelregisteret** er et helseregister med informasjon om reseptbelagte legemidler utlevert i apotek.	x	x	x
Innleggelse for anoreksi eller annen spiseforstyrrelse og/eller psykisk lidelse.	**Norsk pasientregister NPR**	x	x	x
Innleggelse for anoreksi eller annen spiseforstyrrelse og/eller psykisk lidelse.	**KUHR (Kontroll og utbetaling av helserefusjoner)** er et system som håndterer refusjonskrav fra behandlere og helseinstitusjoner til staten (HELFO).	x	x	x

Deling av helseopplysninger

Involverte samarbeidspartnere må signere en gyldig avtale om data og material overføring før data og/eller materiale eventuelt overføres. En slik avtale har som formål å regulere rettigheter og plikter ihht personopplysningsloven og personvernforordningen (datatilsynet).

Deling av helseopplysninger til andre land

Helseopplysninger skal ikke sendes ut av landet.

d) fra hvilke kilder humant biologisk materiale skal uttas og om slikt materiale skal utleveres til andre eller overføres til utlandet

Utover prøveinnsamling som inngår i standard utredning ved innleggelse og behandling i klinikken vil det samles inn ett ekstra serumrør for biobanking og senere analyse av andre biomarkører. Prøven vil deles i 4 allikvoter hvorav 2 vil lagres i Forskningsbiobanken for NORMA studien. Fra kontrollene vil det bli tatt to rør for direkte analyse av kliniske biomarkører hos Fürst samt to serumrør som vil lagres i forskningsbiobank for NORMA studien (se avsnitt om utvidet samtykke). Serumprøvene vil etter planen analyseres ved laboratorier i Norge, fortrinnsvis ved OUS og NMBU.

Avføringsprøvene vil lagres i forskningsbiobank for NORMA studien (se avsnitt om utvidet samtykke). Prøvene vil bli benyttet til biomarkøranalyser og til kartlegging av tarmflora basert på isolert mikrobielt DNA og påfølgende sekvensering. I tillegg prepareres et fekalt transplantat til WP2 og 3 fra et utvalg av pasientprøvene og kontrollene. Primært planlegger vi at laboratoriepreparering av materiale for sekvensering og sekvenseringen skal foregå ved NMBU men dersom det er økonomisk hensiktsmessig å kjøpe denne tjenesten fra eksterne leverandører i Norge eller i utlandet, som for eksempel Oslo sekvenseringssenter, DNAsense (Danmark), Eurofins (Tyskland), Ziel (Tyskland) eller Novogene (UK), ønsker vi å ha mulighet til dette. Ved en eventuell oversendelse til utenlandsk laboratorium vil prøvene kun være merket med anonyme ID-numre og REK vil informeres dersom vi inngår en slik avtale med detaljert beskrivelse av rutine for material- og dataoverføring og avtaler knyttet til dette.

e) vurdering av forskningsetiske utfordringer ved prosjektet, særlig nytte-risiko aspektet for forskningsdeltakere

Utover avføringsinnsamling, et par ekstra blodprøver og kostundersøkelsen/skjemautfylling vil ikke pasientdeltakerne utsettes for noe nevneverdig større belastning enn de utsettes for under oppfølging/behandling ved sengepost for spiseforstyrrelser. Samlet mener vi at nytten av prosjektet står i rimelig forhold til den risiko/ulempe som pasientene utsettes for. For kontrollene er konklusjonen den samme. Kontrollene vil få dypere innsikt i eget kosthold og gode råd for hvordan de kan spise sunnere. Tilbakemelding på de diagnostiske blodverdiene vil også være en nyttig helsesjekk.

Det er flere etiske problemstillinger knyttet til intervensjonsforsøk med mat i anoreksi-populasjoner siden interferens med standardbehandling kan ha uventede og uønskede effekter. Bruken av de prekliniske metoder i det aktuelle prosjektet tar derfor hensyn disse problemstillingene og gjør det mulig å studere tarmfloraen i en ex vivo setting der den eksperimentelt kan manipuleres.

De to foreningene for spiseforstyrrelser, SPISFO og ROS, som er samarbeidspartnere i studien, har deltatt i flere planleggingsmøter for studien og har kommet med innspill til forskningsprotokoll og informasjonsskriv til deltakerne. Dermed har brukerperspektivet blitt grundig ivaretatt i planleggingen av studien.

f) finansieringskilder, interesser og avhengighetsforhold, herunder forskere og forskningsdeltakeres eventuelle økonomiske forhold knyttet til det aktuelle forskningsprosjektet

Prosjektet er finansiert av forskningsrådet (Prosjektnummer 336239). Kontrollene vil informeres om at de vil være med i trekningen om et (av 5) gavekort på 1000 kr. Pasientdeltakerne i studien vil bli kompensert med et gavekort på 250 kr pr måletidspunkt. Pga beløpets beskjedne sum og den lave sannsynligheten for å vinne anser vi at den økonomiske påskjønnelsen ikke vil påvirke avgjørelsen om deltakelse nevneverdig. Ingen av de deltagende forskere har økonomiske interesser av prosjektet.

g) plan for offentliggjøring av resultater og opplysninger om mulig utvidet bruk, herunder kommersiell bruk, av forskningsresultater, data eller biologisk materiale.

Plan for offentliggjøring av resultater: Resultatene fra prosjektet vil først og fremst publiseres som artikler gjennom tradisjonelle vitenskapelige kanaler. De tre første artiklene vil inngå i en PhD grad som er finansiert av bevilgningen fra forskningsrådet. Vi kommer også til å kommunisere resultatene fra studien gjennom den norske mikrobiota organisasjonen ReMicS (https://microbiota.no/remics/) og via websider ved OUS og NMBU. Vi vil lage en egen webside for NORMA studien som driftes av NMBU. De to brukerorganisasjonene vil også bidra med popularisering av resultatene og bidra til effektiv kommunisering av resultatene til sine brukere. I tillegg vil vi sikte mot en bred omtale av publiserte resultater i sosiale medier og TV/radio. Prosjektleder har allerede blitt intervjuet av NRK.no og NRK radio (EKKO) etter tildelingen av forskningsmidler, hatt innlegg på kvinnehelsekonferansen arrangert av Forskningsrådet (desember 2022) og har en stående invitasjon om å intervjues på TV programmet God Morgen Norge for å snakke om prosjektet.

Opplysninger om mulig utvidet bruk: Vi ønsker å beholde mulighet for noe utvidet bruk av data og materiale fremkommet i studien for pasientgruppen og kontrollene i perioden studien varer. Deltakerne i studien blir derfor spurt om de, i tillegg til å være med i NORMA-studien, vil gi et bredt samtykke til at materiale og data inngår i en generell biobank med tilhørende helseregister (GUTBRAIN). Til informasjon søkes det parallelt om opprettelse av GUTBRAIN prosjektet til REK. Formålet med GUTBRAIN er at biomateriale og data fra studier som undersøker tarm-hjerne aksen kan samles for sammenlikning mellom ulike populasjoner utover anoreksi samt til eksperimentelle studier som kan øke forståelsen av tarmfloraens påvirkning på tarm-hjerne-aksen.

1 Steinhausen, H. C. The outcome of anorexia nervosa in the 20th century. *Am. J. Psychiatry* **159**, 1284-1293 (2002).

2 Murray, S. B., Quintana, D. S., Loeb, K. L., Griffiths, S. & Le Grange, D. Treatment outcomes for anorexia nervosa: a systematic review and meta-analysis of randomized controlled trials. *Psychol. Med.* **49**, 535-544, doi:10.1017/S0033291718002088 (2019).

3 Henriksen, H. B. *et al.* Validation of two short questionnaires assessing physical activity in colorectal cancer patients. *BMC sports science, medicine & rehabilitation* **10**, 8, doi:10.1186/s13102-018-0096-2 (2018).

4 La Rosa, P. S. *et al.* Hypothesis testing and power calculations for taxonomic-based human microbiome data. *PloS one* **7**, e52078, doi:10.1371/journal.pone.0052078 (2012).
