# Supplementary material for: The Norwegian Microbiota Study in Anorexia Nervosa (NORMA): Integrating a clinical trial with preclinical experiments–A study protocol
Source: PLoS One. 2026 Mar 11;21(3):e0342275. doi: 10.1371/journal.pone.0342275 (PMC12978472; doi:10.1371/journal.pone.0342275)
Supplement: S4 File — (PDF) [file pone.0342275.s004.pdf]

Supplementary Method Description

The Norwegian Microbiota Study in Anorexia Nervosa (NORMA): integrating a clinical trial with preclinical experiments – a study protocol

Ida Heir Hovland[1,2], Lasse Bang[3], Anne Mari Herfindal[1], Stine Sofie Strømland[1], Tina Bogetvedt Spernes[1], Armita Jahanshahi[1], Kathinka Larsen Otterdal[1], Dunja Arsenovic[1], Trude Elise Aspholm[1], Ylva Vik[1], Jenny HM Storvik[1], Monica Hauger Carlsen[4], Monica Linnea Ones[5], Renata Alisauskiene[5], Kristina Hansen[6], Siri Weider[7,15], Ivan Samdal[7], Johan Dahl[8], Hilde Tveit Reistad[8], Åsne Skram Trømborg[1], Lars Jordhøy Lindstad[1], Signe Birkeland[1], Hanne Torp Eriksen[9], Jeanette Engeset[10], Cynthia M. Bulik[11,12,13], Bjørge Westereng[1], Harald Carlsen[1], Øyvind Rø[2,14] and Siv Kjølsrud Bøhn[1*]

[1]Faculty of Chemistry, Biotechnology and Food Sciences, Norwegian University of Life Sciences, Ås, Norway

[2]The Regional Department for Eating Disorders, Oslo University Hospital, Oslo, Norway

[3]Department of Child Health and Development, Norwegian Institute of Public Health, Oslo, Norway

[4]Department of Nutrition, Institute of Basic Medical Sciences, University of Oslo, Oslo, Norway

[5]The regional Department of Eating Disorders, Haukeland University Hospital, Bergen, Norway

[6]Regional Center for Eating Disorder, Nordland Hospital, Bodø, Norway

[7]Regional Eating Disorder Unit, Nord-Trøndelag Hospital Trust, Levanger, Norway

[8] Research institute of Modum Bad, Vikersund, Norway

[9] District Psychiatric Center, Innlandet Hospital, Gjøvik, Norway

[10]District Psychiatric Center, Søndre Oslo, Norway

[11]Department of Medical Epidemiology and Biostatistics, Karolinska Institutet, Stockholm, Sweden

[12]Department of Psychiatry, University of North Carolina at Chapel Hill, Chapel Hill, USA

[13]Department of Nutrition, University of North Carolina at Chapel Hill, Chapel Hill, USA

[14]Institute of Clinical Medicine, University of Oslo, Oslo, Norway

[15]Department of Psychology, the Norwegian University of Science and Technology (NTNU), Trondheim, Norway

***Corresponding author:** email address: sivb@nmbu.no (SKB)

Sample size calculation for the initial mouse experiment in NORMA WP3

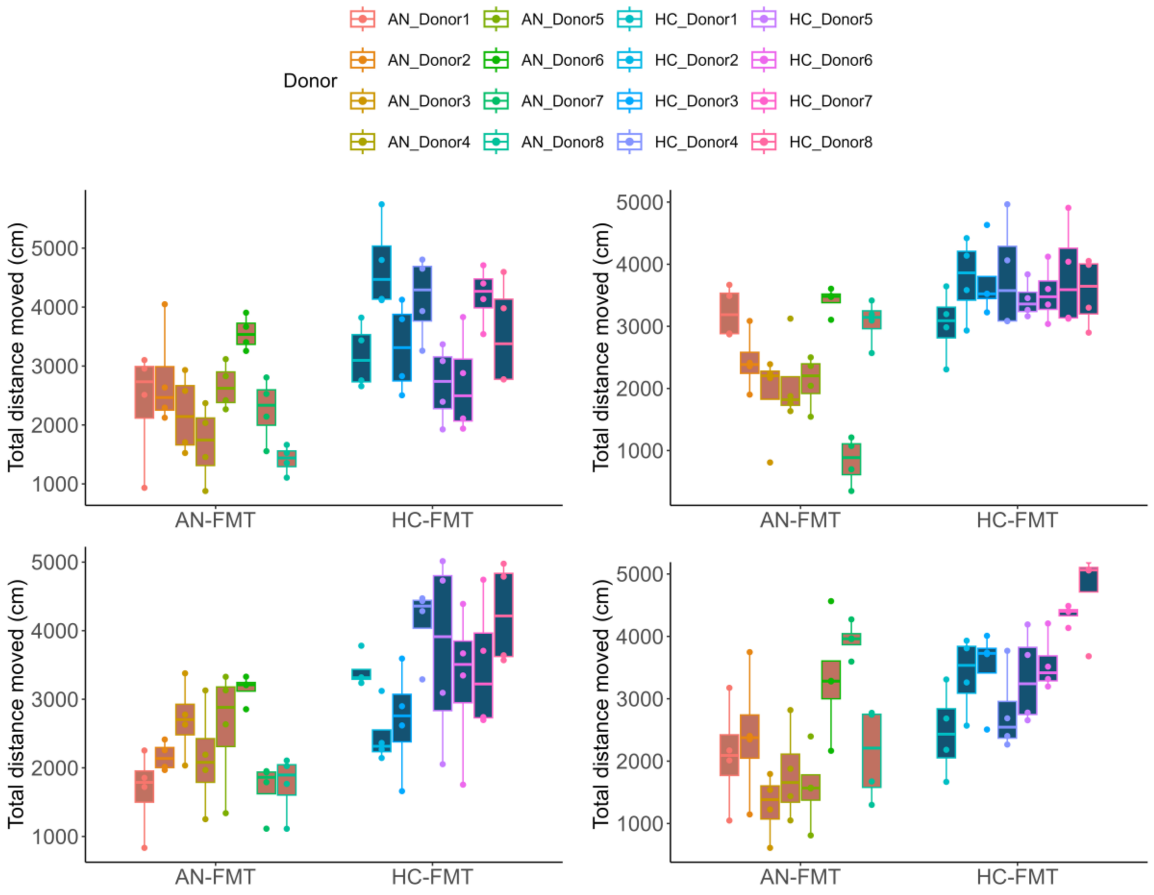

Supplemental Figure 1. Four examples of simulated datasets, all with eight AN donors, eight HC donors, and four mice per donor.

R code #1 (was ran in version 4.4.3): gives an example of how a simulated dataset with eight AN donors, eight HC donors, and four mice per donor was made.

```
#####################################################################
### Function to simulate dataset
###
suppressMessages(suppressWarnings(library(tidyverse)))
generate_dummy_data <- function(n_an_donors, n_hc_donors, n_mice_per_donor,
                                mean_an = 100, mean_hc = 150,
                                sd_donor = 10, sd_within = 15) {

  # Create donor IDs
  an_donors <- paste0("AN_Donor", 1:n_an_donors)
  hc_donors <- paste0("HC_Donor", 1:n_hc_donors)
  donors <- c(an_donors, hc_donors)

  # Create group labels
  donor_groups <- c(rep("AN", n_an_donors), rep("HC", n_hc_donors))

  # Initialize data frame
  data <- data.frame()

  # Generate data for each donor
  for (i in seq_along(donors)) {
    group <- donor_groups[i]
    donor_mean <- ifelse(group == "AN", mean_an, mean_hc) + rnorm(1, 0, sd_donor)
    mice_data <- data.frame(
      Donor = donors[i],
      Group = group,
      # Note:
      # Here we use rtruncnorm() instead of rnorm()
      # Based on previous data from our lab, the maximum deviation from mean was 1490 cm.
      # Therefore, in the generated dataset, a mouse within a donor cannot deviate more than
      # 1700 cm from the donor mean. E.g., if the donor mean is 2000 cm, a mouse within this
      # donor cannot have response lower than 300 cm or higher than 3700.
      Response = rtruncnorm(n = n_mice_per_donor,
                            mean = donor_mean,
                            sd = sd_within,
                            a = donor_mean - 1700,
                            b = donor_mean + 1700)
    )
    data <- rbind(data, mice_data)
  }

  # Note:
  # Here we "force" the difference in AN and HC overall mean
  # to be mean_hc-mean_an
  diff_generated <- data %>% group_by(Group) %>%
    summarise(Mean = mean(Response)) %>%
    pivot_wider(values_from = Mean, names_from = Group) %>%
    mutate(Diff = HC-AN) %>%
    pull(Diff)
  # Negative number, eg. -63 -> the HC mean is 63 LOWER than it "should" compared to AN
  # Positive number, eg. 120 -> the HC mean is 120 HIGHER than it "should"  compared to AN
  diff_adjust <- diff_generated - (mean_hc-mean_an)
  data <- data %>%
    mutate(Response = ifelse(Group == "HC",
                             Response - diff_adjust,
                             Response))
  return(data)
}
```

R code #1 continued.

```
####################################################################
### Define dataset characteristics
###
# Expected mean in the AN-FMT and HC-FMT groups
mean_hc = 3510
mean_an = 3510*0.66

# Expected sd in donor means
Hata_donor_means_HC <- c(4800, 4100, 4000, 3650)
Hata_donor_means_AN <- c(4650, 3650, 3400, 3300)
Hata_donor_sd_HC <- sd(Hata_donor_means_HC)
Hata_donor_sd_AN <- sd(Hata_donor_means_AN)
Hata_donor_sd = ((Hata_donor_sd_HC+Hata_donor_sd_AN)/2)
Hata_mean_HC <- 4200
Hata_donor_sd_percent <- (Hata_donor_sd/4200)
Our_lab_sd_10min <- (749/3510) # sd/mean with 10-min tracking
Our_lab_sd_5min <- (510/2088) # sd/mean with 5-min tracking
donor_sd_scaling <- Our_lab_sd_5min/Our_lab_sd_10min
donor_sd <- Hata_donor_sd_percent*donor_sd_scaling*mean_hc

# Expected sd between mice within donor
sd_within = 749

####################################################################
### Simulate dataset
###
df <- generate_dummy_data(
                          n_an_donors = 8, # How many AN donors in the AN-FMT group?
                          n_hc_donors = 8, # How many HC donors in the HC-FMT group?
                          n_mice_per_donor = 4, # How many mice per donor?
                          mean_an = mean_an,
                          mean_hc = mean_hc,
                          sd_donor = donor_sd,
                          sd_within = sd_within)



####################################################################
### Plot dataset
###
df %>%
  mutate(Group = ifelse(Group == "AN", "AN-FMT", "HC-FMT")) %>%
  ggplot(mapping = aes(x=Group, y=Response, fill=Group, color = Donor)) +
  geom_boxplot()+
  geom_point(position = position_dodge(width = 0.75))+
  theme_classic() +
  theme(legend.position = "none",
        axis.title = element_text(size = 16),
        axis.text = element_text(size = 16),
        plot.title = element_text(size = 16, face = "bold"),
        plot.subtitle = element_text(size = 14))  +
  scale_fill_manual(values = c("#C07161", "#145272")) +
  labs(y = "Total distance moved (cm)",
       x = "")
```

R code #2 (was ran in version 4.4.3): For each of the 30 000 simulated datasets, a linear mixed model was fitted with Group (i.e., AN-FMT vs. HC-FMT) as a fixed effect and donor as a random effect using the following code. Then, a simulation-based power analysis was performed for each of the 30 000 models.

```
####################################################################
### Get the power for the simulated dataset
###
# Fit model
model_df <- lme4::lmer(Response ~ Group + (1|Donor), data=df)
# Perform power calculation
simr::powerSim(model_df,
progress = TRUE,
 nsim = 1000) # OBS, this takes some time.


####################################################################
### Get the P-value for the simulated dataset
###
# Fit model
model1 <- lmerTest::lmer(Response ~ Group + (1|Donor), data=df)
# Get P-value for the group effect
summary(model1)
```

The power results from the 30 000 simulated datasets are summarized in **Supplemental Figure 2** and **Supplemental Table 1** below. The color of the dots in the Figure indicates the p-value for the Group effect in the fitted model while the y-axis shows the simulation-based statistical power for the corresponding dataset. Exact statistics for each combination of donor number and donor group size are provided in **Supplemental Table 1**.

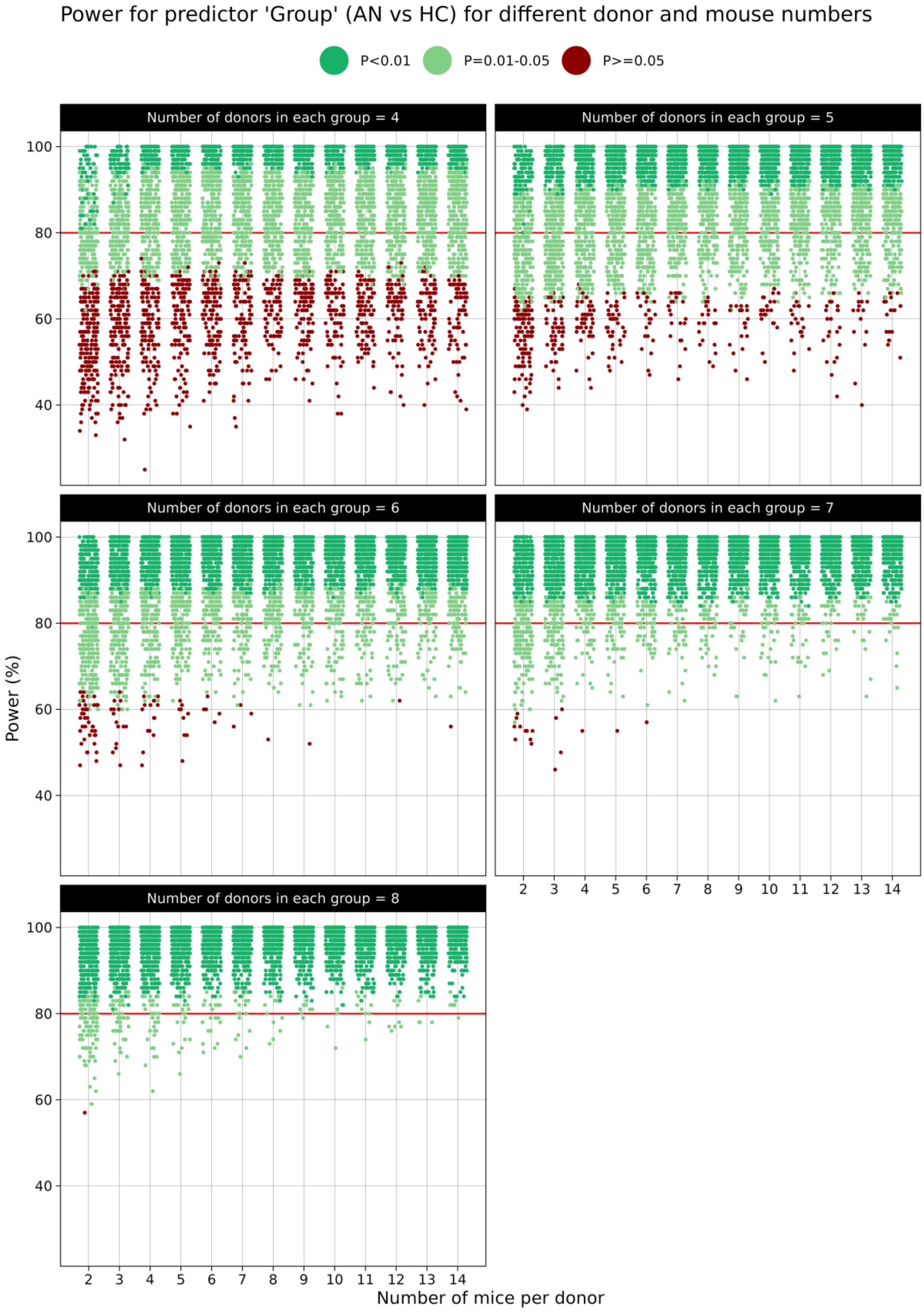

Supplemental Figure 2. Power results from the 30 000 simulated datasets. The color of the dots indicates the p-value for the Group effect in the fitted model while the y-axis shows the simulation-based statistical power for the corresponding dataset.

Supplemental Table 1. Power results from the 30 000 simulated datasets. Exact statistics for each combination of donor number and donor group size.

Number of donors per group	Number of mice per donor	Mean power (%)	Minimum power (%)	Maximum power (%)	Number of datasets with power above 80%		Number of datasets with P-value below 0.05		Number of simulated datasets
					N	%	N	%	
4	2	70	33	100	144	29	274	55	500
4	3	76	32	100	221	44	322	64	500
4	4	79	25	100	269	54	362	72	500
4	5	80	35	100	271	54	364	73	500
4	6	82	41	100	297	59	380	76	500
4	7	83	35	100	315	63	397	79	500
4	8	84	46	100	320	64	412	82	500
4	9	84	41	100	309	62	403	81	500
4	10	85	38	100	336	67	416	83	500
4	11	84	44	100	331	66	406	81	500
4	12	84	40	100	325	65	420	84	500
4	13	84	40	100	329	66	416	83	500
4	14	85	39	100	334	67	422	84	500
5	2	76	39	100	209	42	375	75	500
5	3	82	44	100	290	58	450	90	500
5	4	84	44	100	323	65	450	90	500
5	5	86	48	100	352	70	462	92	500
5	6	87	47	100	368	74	479	96	500
5	7	88	46	100	397	79	478	96	500
5	8	89	49	100	404	81	480	96	500
5	9	89	46	100	392	78	478	96	500
5	10	89	50	100	397	79	478	96	500
5	11	89	48	100	402	80	480	96	500
5	12	90	42	100	407	81	478	96	500
5	13	90	40	100	410	82	485	97	500
5	14	90	51	100	401	80	482	96	500
6	2	82	47	100	288	58	458	92	500
6	3	86	47	100	367	73	483	97	500
6	4	89	47	100	404	81	485	97	500
6	5	91	48	100	421	84	491	98	500
6	6	91	57	100	440	88	495	99	500
6	7	92	56	100	452	90	497	99	500
6	8	92	53	100	447	89	499	100	500
6	9	93	52	100	456	91	499	100	500
6	10	93	62	100	462	92	500	100	500
6	11	93	62	100	459	92	500	100	500
6	12	93	62	100	459	92	499	100	500
6	13	94	63	100	468	94	500	100	500
6	14	93	56	100	457	91	499	100	500
7	2	86	52	100	356	71	490	98	500
7	3	90	46	100	418	84	496	99	500
7	4	93	55	100	465	93	499	100	500
7	5	93	55	100	463	93	499	100	500
7	6	94	57	100	465	93	499	100	500
7	7	94	62	100	472	94	500	100	500
7	8	95	68	100	479	96	500	100	500
7	9	95	63	100	484	97	500	100	500
7	10	95	62	100	478	96	500	100	500
7	11	95	63	100	483	97	500	100	500
7	12	96	68	100	486	97	500	100	500
7	13	96	63	100	493	99	500	100	500
7	14	96	65	100	492	98	500	100	500
8	2	90	57	100	418	84	499	100	500
8	3	94	66	100	469	94	500	100	500
8	4	95	62	100	478	96	500	100	500
8	5	95	66	100	489	98	500	100	500
8	6	96	71	100	489	98	500	100	500
8	7	96	70	100	490	98	500	100	500
8	8	97	73	100	494	99	500	100	500
8	9	97	79	100	498	100	500	100	500
8	10	97	72	100	497	99	500	100	500
8	11	97	74	100	496	99	500	100	500
8	12	97	76	100	495	99	500	100	500
8	13	97	78	100	497	99	500	100	500
8	14	98	79	100	499	100	500	100	500

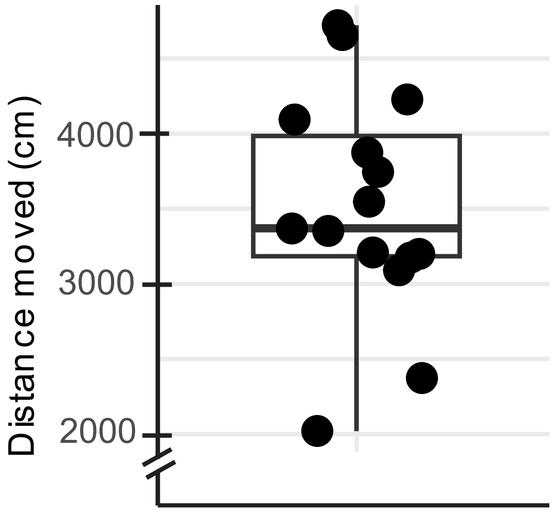

Supplemental Figure 3. The total distance moved (cm) throughout a 10-minute period of tracking for 15 C57BL/6JRj female mice (age 19 weeks) in the Open field test. The boxplot shows the median (center line), interquartile range (box), and whiskers indicating the minimum and maximum values. Individual data points are shown as jittered dots. The mice were tested as part of a pilot study to obtain an empirical estimate of outcome variability for use in sample size calculations. Mean (SD) total distance moved was 3510 cm (749 cm). The mice were tested under the same experimental conditions as planned for the main study, including housing, handling procedures, testing environment, and open field apparatus settings.
